# Efp promotes *in vitro* and *in vivo* growth of endometrial cancer cells along with the activation of nuclear factor-κB signaling

**DOI:** 10.1371/journal.pone.0208351

**Published:** 2018-12-26

**Authors:** Wataru Sato, Kazuhiro Ikeda, Tomohiko Urano, Yayoi Abe, Norie Nakasato, Kuniko Horie-Inoue, Satoru Takeda, Satoshi Inoue

**Affiliations:** 1 Division of Gene Regulation and Signal Transduction, Research Center for Genomic Medicine, Saitama Medical University, Hidaka-shi, Saitama, Japan; 2 Department of Geriatric Medicine, Graduate School of Medicine, The University of Tokyo, Bunkyo-ku, Japan; 3 Department of Obstetrics and Gynecology, School of Medicine, Juntendo University, Bunkyo-ku, Tokyo, Japan; 4 Department of Obstetrics and Gynecology, Saitama Medical Center, Saitama Medical University, Kawagoe-shi, Saitama, Japan; 5 Functional Biogerontology, Tokyo Metropolitan Institute of Gerontology, Itabashi-ku, Tokyo, Japan; University of South Alabama Mitchell Cancer Institute, UNITED STATES

## Abstract

Endometrial cancer is common among postmenopausal women and its incidence is increasing in developed countries. Considering that >80% of endometrial cancers are assumed to be estrogen-related, higher estrogen exposure will be relevant to tumorigenesis. Therefore, the roles of estrogen target genes will be important to understand the pathophysiological mechanisms. We previously revealed that estrogen-responsive RING finger protein Efp contributes to breast cancer progression through the protein degradation of cell cycle checkpoint 14-3-3σ. We and others also proposed that Efp has tumor-promoting activities in estrogen receptor (ER)-negative cancer cells. In addition, Efp plays a role in type I interferon production by activating antiviral signaling, which provokes nuclear factor-κB (NF-κB) signaling. In the present study, we investigate whether Efp plays a critical role in endometrial cancer biology. We show that siRNA-mediated Efp knockdown represses the proliferation and migration of endometrial cancer ER-positive Ishikawa and ER-negative HEC-1A cells. Efp knockdown increases 14-3-3σ protein levels and decreases the rates proliferative stage cells. Efp siRNA significantly inhibits the *in vivo* tumor growth of endometrial cancer cells in both subcutaneous and orthotopic xenograft models. Intriguingly, Efp knockdown represses NF-κB-dependent transactivation and transcription of target genes, such as *IL6ST* and *IL18*, in endometrial cancer cells. Overall, Efp would exert a tumor-promoting role through modulating NF-κB pathway and 14-3-3σ protein degradation in endometrial cancer regardless of its estrogen receptor status. Our results indicate that Efp could be a potential diagnostic and therapeutic target for endometrial cancer.

## Introduction

Endometrial cancer is one of the most common cancers in women. In terms of clinical and pathological features, endometrial cancers are classified into two major types, type I and II. Approximately 80–90% of endometrial cancers are known as type I. Type I endometrial cancer is typically low-grade adenocarcinoma that is referred to as estrogen-related because it is assumed to be developed in response to prolonged and unopposed estrogen stimulation [1]. Type II endometrial cancer has non-endometrioid histology and mainly occur in postmenopausal women and thus the subtype is considered as estrogen-independent [2]. From these clinical situations, the majority of endometrial cancers likely associate with estrogen signaling, so higher estrogen exposure due to such as obesity seems to increase disease incidence. Nevertheless, endocrine therapy is not widely used to treat patients with endometrial cancer because of its limited effect [3]. Under these circumstances, understanding the pathophysiology of endometrial cancer would facilitate the development of new therapeutic options for the disease.

We previously identified Efp as an estrogen target gene based on genomic binding-site cloning [4]. Efp protein harbors a tripartite motif (TRIM) structure composed of RING finger, B box, and coiled coil domains, and these conserved domains are usually observed in prototypic proteins of TRIM superfamily. These conserved domains are found in the superfamily of TRIM proteins [5]. In various clinical cancers, Efp has been shown to contribute to the aggressiveness of the diseases. In breast cancer cells, Efp is demonstrated to act as an E3 ubiquitin ligase that targets cell cycle checkpoint protein 14-3-3σ for the proteasomal degradation, leading to the promotion of tumor formation in a mouse xenograft model [6]. Clinicopathological studies have showed that Efp immunoreactivity correlates with poor prognosis of breast cancer patients with both ER-positive and -negative cancers [7]. Further investigation suggested an additional mechanism for the role of Efp in breast cancer cells. In the presence of estrogen, Efp stimulates the ubiquitination of ERα protein, leading to the enhancement of transcriptional activity by increasing the interaction with transcriptional coactivators while simultaneously targeting ERα for protein degradation [8]. It has been also reported that Efp ubiquitinates and promotes to degrade estrogen-inducible tumor suppressor AT-binding transcription factor 1 (ATBF1) and acts as a negative regulator for ERα-mediated transcription [9]. In addition, estrogen stimulates the degradation of KLF5 protein, which has been identified as an essential cofactor for the tumor suppressor TGF-β, by inducing the expression of Efp [10]. Notably, an integrated systems biology approach identified that Efp is a key determinant of breast cancer metastasis in a recent report [11]. These observations suggest that Efp has a pivotal role in ER-positive and -negative breast cancers.

We previously showed that siRNA-mediated knockdown of Efp efficiently suppressed *in vitro* proliferation and cell-cycle progression of breast cancer cells, and significantly inhibited tumor formation of xenografted breast cancer cells in athymic mice [12]. Therefore, Efp was defined as a critical factor in breast cancer proliferation and could be a novel target of cancer therapy.

Studies for the innate immune system revealed that Efp is an interferon (IFN) responsive gene and modulates the nuclear factor-κB (NF-κB) pathway [13, 14]. For RNA viral infections, Efp induces the lys63-linked ubiquitination of retinoic acid-inducible gene I product (RIG-I), which elicits host antiviral innate immunity. RIG-I then transmits a signal leading to the activation of interferon regulatory factor-3 (IRF-3) and NF-κB to induce interferon-β (IFN-β) and antiviral cytokine gene expression. NF-κB is also involved in cancer initiation, development, metastasis, and resistance to treatment [15]. In a large number of tumors including endometrial cancer, NF-κB is activated due to the inflammatory microenvironment and various oncogenic mutations [16]. The precise role of Efp-mediated NF-κB signaling in cancers, however, remains to be studied.

In normal uterus, Efp is primarily inducible by estrogen treatment [17]. Moreover, Efp knockout mice exhibit underdeveloped uteri and reduced estrogen responsiveness [18]. Considering that the majority of endometrical cancers are estrogen-related, we questioned whether Efp could also play a critical role in the pathophysiology of endometrial cancer. In the present study, we show that siRNAs specifically targeting Efp significantly inhibit the *in vitro* cell growth, cell cycle progression, and migration of endometrial cancer ER-positive Ishikawa and ER-negative HEC-1A cells. In a subcutaneous xenograft tumor model using athymic mice, direct injection of Efp-targeting siRNA into generated tumors suppressed the tumor growth derived from endometrial cancer cells. Moreover, intravenous administration of Efp-targeting siRNA repressed the tumor growth of endometrial cancer cells in an orthotopic xenograft tumor model. In addition, Efp-targeting siRNA decreased NF-κB-mediated transcription and expression of downstream genes. Taken together, we consider that Efp is a critical factor that promotes the proliferation of endometrial cancer by exerting protein degradation of 14-3-3σ as well as by modulating NF-κB signaling.

## Materials and methods

### Cell culture

Human endometrial cancer Ishikawa cells (Ishikawa cells 3H12 No.74) were kindly provided by Dr. Masato Nishida (Kasumigaura Medical Center, Ibaraki, Japan). Human endometrial cancer HEC-1A cells and embryonic kidney 293T cells were obtained from American Type Culture Collection (Rockville, MD, USA). Ishikawa and HEC-1A cells were originally established from a well-differentiated (G1) and moderately differentiated (G2) endometrial adenocarcinoma, respectively [19]. We confirmed the ERα status of Ishikawa (ERα-positive) and HEC-1A (ERα-negative) cells by qRT-PCR as described elsewhere [20]. Cells were maintained in Dulbecco’s modified Eagle’s medium (DMEM) supplemented with 10% fetal bovine serum, penicillin (100 units/mL), and streptomycin (100 μg/mL) at 37°C in a humidified atmosphere of 5% CO_2_ in air. HEC-1A cells were transfected with a luciferase expression plasmid, which was generated by inserting a luciferase gene from the pGL3-basic plasmid (Promega, Madison, WI, USA) into the pCXN2 vector [21], and then stable transformants (HEC-1A-luc) were selected with G-418.

### Transfection of siRNAs and western blot analysis

The siRNA duplexes targeting Efp (siEfp #A and #B), ERα (siERα #A and #B), and a non-targeting control siRNA (siControl) were synthesized by RNAi Inc. (Tokyo, Japan). The sense and antisense strands of Efp, ERα, and control siRNAs were as follow: siEfp #A, 5′-GGGUGGGCGUGCUUCUCAACU-3′ (sense) and 5′-UUGAGAAGCACGCCCACCCGC-3′ (antisense); siEfp #B, 5′-GGGAUGAGUUCGAGUUUCUGG-3′ (sense) and 5′-AGAAACUCGAACUCAUCCCUC-3′ (antisense); siERα #A, 5′-GCCUGGUCAGAUUACGUAUGC-3′ (sense) and 5′-AUACGUAAUCUGACCAGGCCC-3′ (antisense); siERα #B, 5′-GGGAGCGUGAUCUAGAUUACA-3′ (sense) and 5′-UAAUCUAGAUCACGCUCCCAA-3′ (antisense); siControl, 5′-GUACCGCACGUCAUUCGUAUC-3′ (sense) and 5′-UACGAAUGACGUGCGGUACGU-3′ (antisense). Cells were plated in 6-well plates at a density of 1 × 10^5^ cells/well and transfected with siRNA at a final concentration of 10 nM using RNAiMAX (Invitrogen, Carlsbad, CA, USA). After 48 h, cell lysates were prepared in a sample buffer for sodium dodecyl sulfate-polyacrylamide gel electrophoresis (SDS-PAGE), heated at 100°C for 15 min, and subjected to SDS-PAGE and western blotting analysis using antibodies for Efp, 14-3-3σ, and β-actin as described previously [12].

### Quantitative PCR analysis

Total RNAs were extracted from the cells and the tumors generated in athymic mice by using Isogen reagent (Nippongene, Tokyo, Japan). Real-time quantitative reverse transcriptase (RT)-PCR (qPCR) was performed according to a method described in a previous report [12]. Briefly, first-strand cDNA was synthesized from 1 μg of total RNA by using SuperScript III reverse transcriptase (Invitrogen) and oligo(dT)20 primer. The mRNA was quantified by real-time PCR using the KAPA SYBR Fast 2X qPCR Master Mix (KAPA Biosystems, Boston, MA, USA) and the StepOne Plus real-time PCR instrument (Applied Biosystems, Foster City, CA, USA) based on SYBR Green I fluorescence. The sequences of the PCR primers were as follows (forward and reverse, respectively): *Efp*, 5′-CAGGAGCTCACCCCCAGTT-3′ and 5′-TTCACAGGGCGTGTGGATT-3′; *ERα*, 5′-AGACGGACCAAAGCCACTTG-3′ and 5′-CCCCGTGATGTAATACTTTTG-3′; *IL6ST*, 5′-CCGTCAGTCCAAGTCTTCTCAA-3′ and 5′-GCCGCTCCTCTGAATCTAACA-3′; *IL18*, 5′-TGCACCCCGGACCATATTTA-3′ and 5′-CTTCACAGAGATAGTTACAGCCATACCT-3′, and *36B4* 5′-CCACGCTGCTGAACATGCT-3′ and 5′-GATGCTGCCATTGTCGAACA-3′. The relative amount of PCR product was calculated by the comparative cycle threshold (CT) method, using *36B4* as an endogenous reference gene.

### Cell proliferation assay

Cell proliferation was assessed in terms of the cell viability by using a kit containing 2-(2-methoxy-4-nitrophenyl)-3-(4-nitrophenyl)-5-(2,4-disulfophenyl)-2H-tetrazolium, monosodium salt (WST-8) (Nacalai Tesque, Kyoto, Japan) [12]. Ishikawa and HEC-1A cells were seeded on 96-well plates at a density of 4 × 10^3^ cells/well and transfected with siRNAs targeting Efp or ERα, or negative control siRNA (siControl) by using Lipofectamine RNAiMAX transfection reagent (Invitrogen). At the indicated time points after the transfection, 10 μL of a reagent solution containing WST-8 was added to each well, and the cells were incubated for 2 h at 37°C. The absorbance of the plates was read on a microplate reader at a wavelength of 450 nm.

### Cell-cycle analysis

Ishikawa and HEC-1A cells were transfected with siRNAs at a final concentration of 10 nM for 48 h and fixed in 70% ethanol. They were then washed twice with PBS and treated with 0.8 mg/mL RNase A for 30 min. Finally, they were stained with 10 μg/mL propidium iodide. The samples were sorted based on DNA content by using fluorescence-activated cell sorting (FACS) (FACScalibur; Becton Dickinson, Cockeysville, MD, USA) and the CellQuest software (Becton Dickinson) in order to determine the percentages of cells that were in the G1, S, and G2/M phases of the cell cycle [12].

### Cell migration assays

Ishikawa and HEC-1A cells were transfected with siRNAs at a final concentration of 10 nM for 24 h. Cells were seeded on a polycarbonate membrane insert in a transwell apparatus (Costar, Cambridge, MA, USA) at a concentration of 1 × 10^5^ cells. After the cells were incubated for 48 h at 37°C in a 5% CO_2_ atmosphere, the migrated cells were fixed with methanol, stained with Giemsa solution, and counted under a microscope in five predetermined fields (200×).

### Microarray analysis

Ishikawa cells were treated with siEfp #A or siControl for 48 h. Total RNA was isolated from the cells using ISOGEN and subjected to microarray analysis using the GeneChip Human Exon 1.0 ST Array (Affymetrix, Santa Clara, CA, USA) according to the manufacturer’s instructions. All microarray data are available in the Gene Expression Omnibus (GEO) database with the accession number GSE118228. A computational approach to evaluate the microarray data at the level of gene sets was performed by employing Gene Set Enrichment Analysis (GSEA) [22].

### Subcutaneous and orthotopic xenograft models of endometrial cancer cells

All animal experiments were approved by the Animal Care and Use Committee of Saitama Medical University (Permit Number: 2041), and conducted in accordance with the Guidelines and Regulations for the Care and Use of Experimental Animals by Saitama Medical University. Female athymic mice (BALB/cAJcl-nu/nu) were purchased from Clea Japan Inc. (Tokyo, Japan) and allowed free access to standard chow and water in a specific pathogen-free mouse facility. Acclimatization period of at least 1 week was taken before the animal experiments. All efforts were made to minimize animal suffering and inhalational anesthesia with isoflurane (5% in 2 L/min. oxygen) was performed at surgical operation and administration of siRNA. At the experimental endpoint, animals were sacrificed by cervical dislocation under isoflurane anesthesia.

For the subcutaneous xenograft model, Ishikawa cells (5 × 10^5^ cells) mixed with Matrigel matrix (Becton Dickinson) were injected into the flanks of 8-week-old BALB/cAJcl-nu/nu mice, respectively (*n* = 10). The tumors were measured in 2 dimensions with micrometer calipers, and the tumor volume was estimated according to the formula: 0.5 × [(smallest diameter)^2^ × (longest diameter)]. When the average tumor volume exceeded 150 mm^3^, siRNA duplexes (5 μg) and 4 μL GeneSilencer reagent (Gene Therapy System, San Diego, CA, USA) dissolved in 50 μL DMEM were directly injected into the tumors twice a week. At the endpoint of the experiment, the tumors were dissected from the mice, homogenized in a sample buffer for SDS-PAGE, and subjected to western blot analysis with anti-Efp, anti-14-3-3σ, and anti-β-actin antibodies [12].

Orthotopic endometrial tumor inoculation was performed as described elsewhere [23]. Briefly, 11-week-old female athymic mice were anesthetized with isoflurane, and the right uterine horn was pulled out. HEC-1A-luc cells (4 × 10^6^ cells/50 μL PBS) was then injected into the lumen of the uterine horn. The siEfp #A or siControl was prepared with Invivofectamine (Invitrogen) according with the manufacturer’s instruction. Shortly, 100 μg siRNA was mixed with 100 μL Invivofectamine by vortexing, and incubated for 30 min at room temperature. Next, 15 volumes of 5% glucose was added to the mixture and then concentrated by centrifugation using Amicon Ultra-15 centrifugal device (Millipore, Billerica, MA, USA). The volume of the retentate containing the siRNA/Invivofectamine complex was adjusted to 200 μL with 5% glucose and intravenously injected into the mice every 4 days. The mice were injected intraperitoneally with D-luciferin (3 mg/mouse), anesthetized for 5 min with isoflurane, and imaged for 2 min under anesthesia using the *in vivo* imaging system (Photon Imager; Biospace Lab, Paris, France). *In vivo* imaging data were analyzed using the Photo Vision software (BioSpace) for data postprocessing.

### Luciferase assay

Ishikawa and HEC-1A cells at a density of 1 × 10^4^ cells per well on 24-well plates were transfected with 0.34 μg of the NF-κB reporter plasmid pNF-κB (Stratagene, La Jolla, CA) and 0.06 μg pRL-CMV (Promega). At the same time, the cells were also transfected with 0.01 μg Flag-tagged Efp expression plasmid or empty vector (pcDNA3, Invitrogen), and 10 nM siEfp #A and #B or siControl. Forty-eight hours after the transfection, a luciferase assay was performed using the Dual-Luciferase Reporter Assay System (Promega). Data were represented as the mean ± SD of three independent experiments.

### Statistical analysis

Differences between the 2 groups were analyzed using the Student’s *t* test, whereas the differences in the mean values among multiple groups were analyzed using a two-way ANOVA followed by Tukey–Kramer HSD post hoc test. All *P*-values were based on two-tailed statistical analyses, and *P*-values <0.05 were considered to be statistically significant (*, *P* < 0.05 and **, *P* < 0.01).

## Results

### Efp silencing increases 14-3-3σ protein levels in endometrial cancer cells

**Fig 1:**
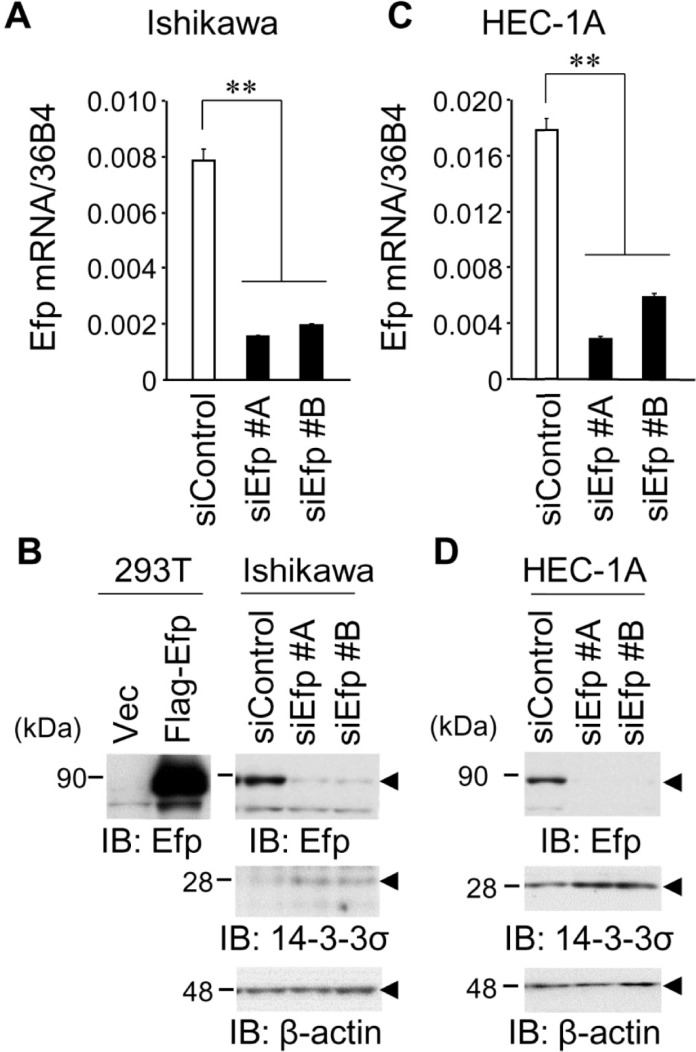
Efp silencing increases 14-3-3σ protein levels in endometrial cancer cells. (**A**) Knockdown efficiency of Efp siRNAs in Ishikawa cells. Cells were transfected with siEfp #A, #B, or negative control siControl for 48 h. *Efp* mRNA levels were determined by qRT-PCR and normalized to *36B4* levels. (**B**) Efp siRNAs decreased Efp protein levels and increased 14-3-3σ protein levels in Ishikawa cells. Whole cell lysates were prepared from Ishikawa cells transfected with siEfp #A, #B, or siControl, and then subjected to western blot analysis using antibodies for Efp and 14-3-3σ. β-actin levels were analyzed as a loading control. (**C**) Knockdown efficiency of Efp siRNAs in HEC-1A cells. Experiments were performed using HEC-1A cells as in (A). Data are presented as means ± s.d. (*n* = 3). **, *P* < 0.01. (**D**) Efp siRNAs decreased Efp protein levels and increased 14-3-3σ protein levels in HEC-1A cells. Experiments were performed using HEC-1A cells as in (B).

To assess the function of Efp in endometrial cancer cells, siEfps (siEfp #A and #B) were introduced both into ERα-positive Ishikawa and ERα-negative HEC-1A endometrial cancer cells. These siRNAs were verified to substantially suppress Efp mRNA and protein expression by qRT-PCR (Fig 1A and 1C) and western blot analysis (Fig 1B and 1D), respectively, both in Ishikawa and HEC-1A cells. Furthermore, since the 14-3-3σ protein has been reported as Efp ubiquitin ligase substrate, the effects of siEfps on 14-3-3σ protein expression were examined. Western blot analysis demonstrated that siEfp #A and #B increased the expression levels of the 14-3-3σ protein in both Ishikawa and HEC-1A cells.

### Efp silencing inhibits growth and cell-cycle progression in endometrial cancer cells

**Fig 2:**
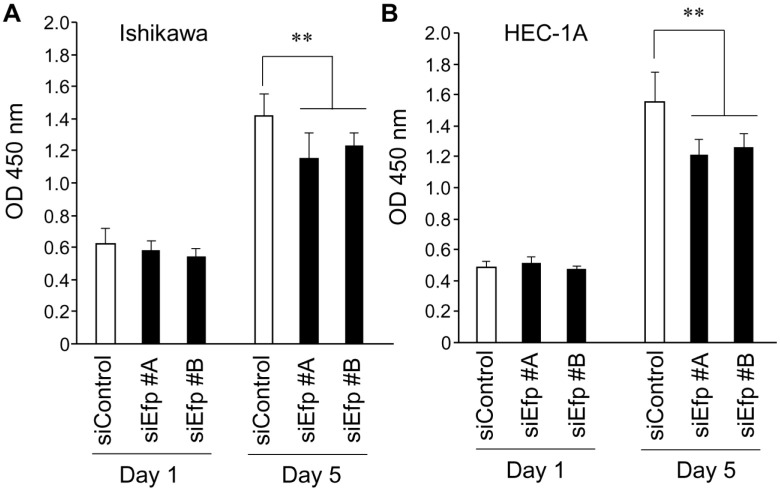
Growth inhibition of endometrial cancer cells transfected with Efp siRNAs. (**A**) Growth inhibition of Ishikawa cells by Efp siRNAs. Ishikawa cells were transfected with siEfp #A, #B, or siControl. The WST-8 cell proliferation assay was performed at the indicated time points after the transfection. The absorbance of the plates was read on a microplate reader at 450 nm. (**B**) Growth inhibition of HEC-1A cells by Efp siRNAs. Experiments were performed using HEC-1A cells as in (A). Data are presented as means ± s.d. (*n* = 3). **, *P* < 0.01.

Next, the effects of siEfps on the proliferation of Ishikawa and HEC-1A cells were examined using WST-8, which is reduced by dehydrogenases in metabolically active cells and then produces a water-soluble colored formazan (Fig 2A and 2B). The Ishikawa and HEC-1A cells proliferated normally in the control conditions with siControl, whereas their proliferation were decreased by treatment with siEfp #A and #B. We evaluated the effects of ERα-targeted siRNAs (siERα #A and #B) on Efp siRNA-dependent cell growth inhibition in ER-positive Ishikawa cells (S1 Fig). The siERα #A and #B repressed the growth of Ishikawa cells to some extent in the presence of estrogen. The extent of cell growth inhibition by Efp siRNAs, however, was substantially greater than that by ERα siRNAs even in the presence of estrogen. Thus we assume that the Efp-mediated proliferation of endometrial cancer cells rather relies on the signaling pathways other than ERα signaling, even in ERα-positive cancer cells.

**Fig 3:**
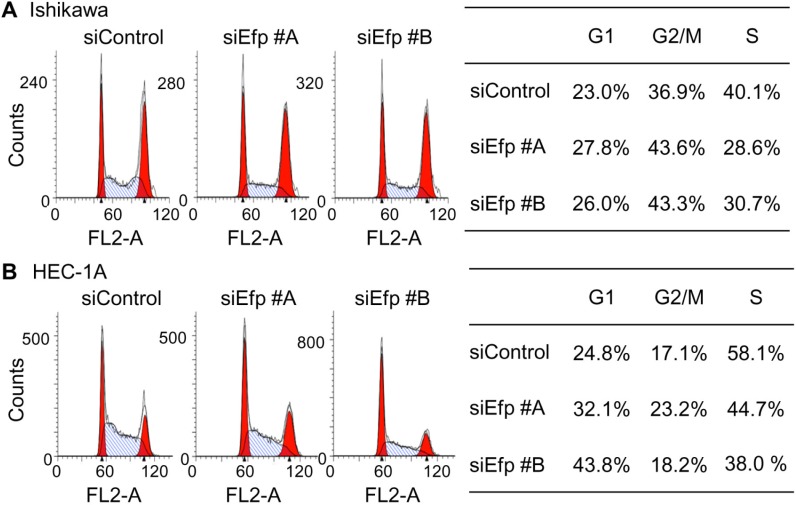
Inhibition of cell-cycle progression in endometrial cancer cells by Efp siRNAs. (**A**) Efp siRNAs decreased the population of Ishikawa cells in S phase. Cells were transfected with siEfp #A, #B, or siControl for 48 h, stained with propidium iodide, and then subjected to FACS analysis. The percentages of cells in the S, G1, and G2/M phases were calculated using the CellQuest software. (**B**) Efp siRNAs decreased the population of HEC-1A cells in S phase. Experiments were performed using HEC-1A cells as in (A).

To determine the effects of Efp siRNA on cell-cycle progression, we performed FACS analysis on Ishikawa cells transfected with the siEfps or siControl (Fig 3A). The percentage of cells in the S phase was decreased by treatment with siEfp #A (28.6%) and #B (30.7%), whereas that of cells in the G1 phase increased by treatment with siEfp #A (27.8%) and #B (26.0%), as compared to the results obtained after transfection with siControl (40.1% and 23.0% for S and G1 phases, respectively). Similarly, in HEC-1A cells, the population of cells in the S phase was decreased by treatment with siEfp #A (44.7%) and #B (38.0%), whereas that of cells in the G1 phase increased by treatment with siEfp #A (32.1%) and #B (43.8%), as compared to the results obtained after transfection with siControl (58.1% and 24.8% for S and G1 phases, respectively) (Fig 3B). These results indicated that Efp siRNAs attenuate the proliferation of Ishikawa and HEC-1A cells.

### Efp silencing suppresses cell migration in endometrial cancer cells

**Fig 4:**
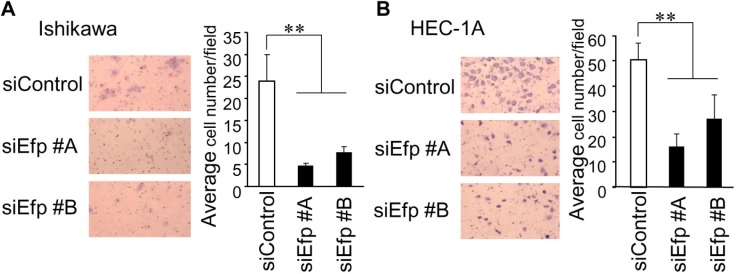
Efp siRNAs suppress the migration of endometrial cancer cells. (**A**) Silencing of Efp decreases migration in Ishikawa cells. Cells transfected with siEfp #A, #B, or siControl were seeded onto a transwell membrane. Migrating cells were fixed and stained with Giemsa. Representative images of migrating cells are shown (magnification, 200×). For each experiment, migrating cells were counted in five random fields (*n* = 3). (**B**) Silencing of Efp resulted in decreased migration in HEC-1A cells. Experiments were performed using HEC-1A cells as in (A). Representative images of migrating cells are shown (magnification, 200×). Data are presented as means ± s.d. (*n* = 3). **, *P* < 0.01.

To further analyze whether Efp plays a significant role in cell migration, siEfp #A and #B were transfected into Ishikawa and HEC-1A cells, and their effects on cell migration were evaluated by a transwell chamber assay (Fig 4A and 4B). The siEfp #A treatment decreased the percentage of migratory cells to 80% and 70% in Ishikawa and HEC-1A cells, respectively, compared with those treated with the siControl. Similarly, siEfp #B treatment decreased the migratory cells to 70% and 60% in Ishikawa and HEC-1A cells, respectively, compared with those treated with the siControl. The results indicated that Efp silencing significantly decreased the migratory potential of both cells.

### Efp silencing suppresses *in vivo* growth of Ishikawa cells

**Fig 5:**
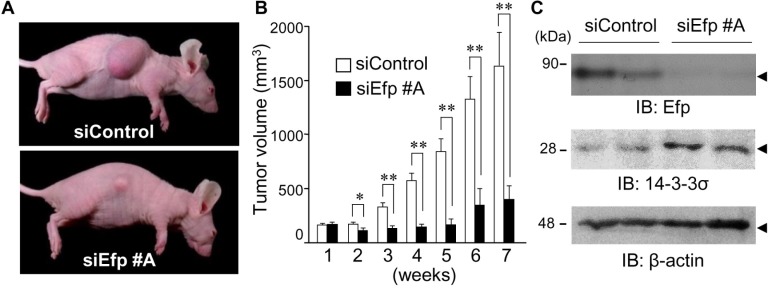
The siEfp treatment suppresses the growth of Ishikawa cell-derived tumors in athymic mice. (**A–C**) Inhibition of tumor formation in athymic mice after Efp siRNA treatment. Eight-week-old female athymic mice were subcutaneously injected with Ishikawa cells (5 × 10^5^ cells) mixed with Matrigel. When the average tumor volume exceeded 150 mm^3^, 5 μg of siEfp #A or siControl mixed with 4 μL of GeneSilencer, a transfection reagent, was directly injected into the formed tumors twice a week. Representative tumors generated in athymic mice are shown (A). The tumor size was measured every week. Data are presented as means ± s.e. (*n* = 10). *, *P* < 0.05; **, *P* < 0.01 (B). Tumors were dissected from the mice 7 weeks after siRNA administration and their homogenates were subjected to western blot analysis using antibodies for Efp and 14-3-3σ. β-actin was analyzed as a loading control (C).

To determine the effect of siEfp on the *in vivo* growth of endometrial cancer cells, we employed a xenograft model in which athymic female mice were subcutaneously inoculated with Ishikawa cells. Either siEfp #A or siControl (5 μg each) mixed with GeneSilencer transfection reagent was directly injected into the tumors every 3 or 4 days after the average tumor volume reached 150 mm^3^. The tumor size was measured just before the time of siRNA injection. At 7 weeks after the siControl injection, substantial tumors developed from the inoculated Ishikawa cells, and the average tumor volume was 1600 mm^3^. In contrast, the tumor volume was almost unchanged by the siEfp #A injection: the average tumor volume at 7 weeks was 300 mm^3^ (Fig 5A and 5B). At the end-point, the protein expression levels of Efp and 14-3-3σ in the generated tumors were determined by western blot analysis (Fig 5C). Cell lysates prepared from tumors treated with siEfp #A contained obviously decreased amounts of Efp protein but increased amounts of 14-3-3σ protein, compared to that of siControl-treated tumors. These results indicated that siEfp #A effectively inhibits tumor growth of Ishikawa cells in mice by specifically repressing Efp expression in the cells.

### Intravenously administered siEfp suppresses endometrial tumor growth in orthotopic mouse model

**Fig 6:**
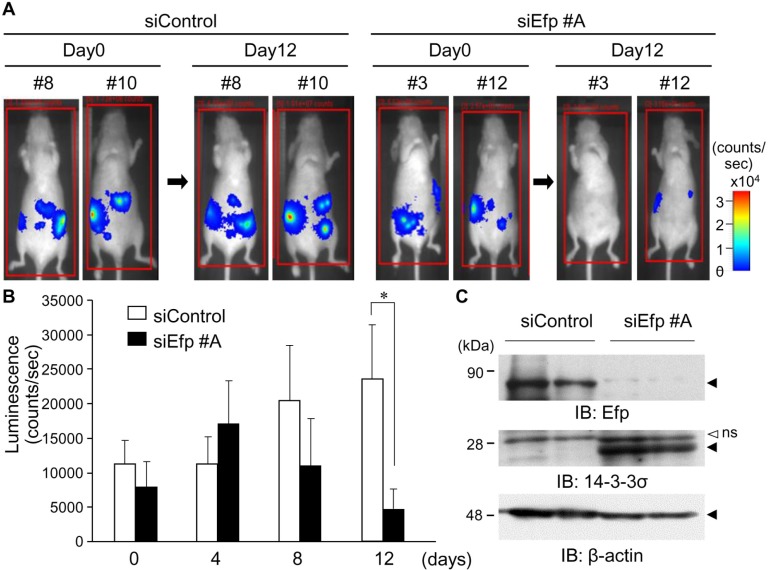
The siEfp treatment suppresses the growth of endometrial cancer cells in the orthotopic xenograft tumor model. (**A–C**) Inhibition of orthotopic tumor formation in athymic mice after Efp siRNA treatment. HEC-1A-luc cells stably transfected with luciferase (4 × 10^6^ cells) were inoculated into the uterus of 11-week-old female athymic mice. The siEfp #A or siControl treatments were prepared with the Invivofectamine transfection reagent and injected to the mice via the tail vein every 4 days (100 μg siRNA complexed with 100 μL Invivofectamine in 200 μL per mouse). The mice were injected intraperitoneally with D-luciferin (3 mg/mouse) and imaged for 2 min under anesthesia using an *in vivo* imaging system (Photon Imager). Representative images are shown (A). Quantification of the signal emitted from HEC-1A-luc cells were performed using the Photo Vision software for data post-processing. Data are presented as means ± s.e. (*n* = 7). *, *P* < 0.05 (B) Tumors were dissected from the mice 12 days after siRNA treatment and their homogenates were subjected to western blot analysis. Efp and 14-3-3σ expression levels were determined using antibodies for Efp and 14-3-3σ. β-actin was analyzed as a loading control (C).

We further examined the tumor suppressive effect of siEfp #A in an orthotopic xenograft model. To achieve this, HEC-1A cells stably transfected with luciferase (HEC-1A-luc) were orthotopically inoculated into the right uterine horn of athymic mice. Tumor growth of HEC-1A-luc cells was estimated by monitoring the intensity of bioluminescence. At the inoculation, the luminescence signal was detected in all cases, whereas the signals were nearly gone after the following day. The signals were, however, detected again 1 month later and increased stably, indicating the sustainable growth of inoculated tumors. These successfully xenografted tumors were used for further examination. Using this orthotopic mouse model, we investigated the effect of intravenously injected siEfp #A on the *in vivo* growth of the HEC-1A-luc cells. The siEfp #A treatment significantly decreased the photon emission by 12 days after injection whereas the siControl treatment did not (Fig 6A and 6B). In this model, some mice had tumors with peritoneal dissemination. In cell lysates prepared from the disseminated tumors in siEfp #A-treated mice, Efp protein levels were remarkably reduced, whereas 14-3-3σ protein levels were increased, compared with that of siControl-treated mice (Fig 6C).

### Efp regulates NF-κB-mediated transcription in endometrial cancer cells

**Fig 7:**
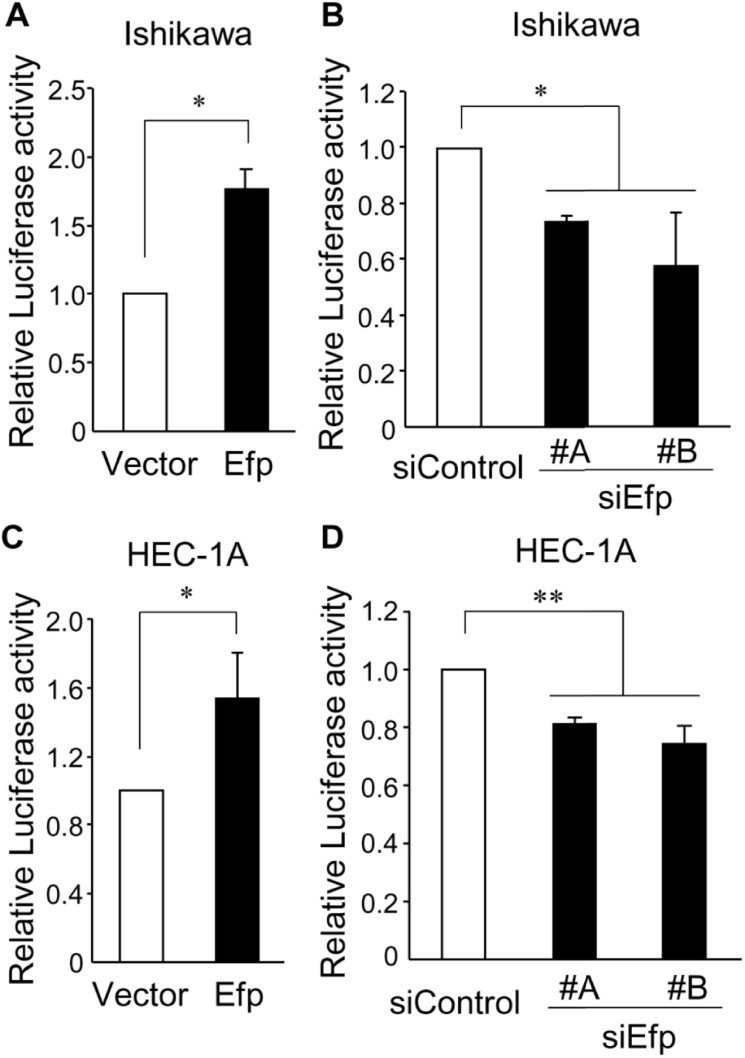
Efp regulates NF-κB-mediated transcription in endometrial cancer cells. (**A**–**D**) Ishikawa (A and B) and HEC-1A (C and D) cells were transfected with NF-κB-Luc and pRL-CMV. At the same time, these cells were also transfected with Flag-Efp (A and C**)** or siEfp #A or #B (B and D). After transfection, cell lysates were subjected to a luciferase assay. Data are presented as means ± s.d. (*n* = 3). *, *P* < 0.05; **, *P* < 0.01.

Since Efp has been shown to have an effect on NF-κB signaling for the recognition of RNA viruses [13, 14], we hypothesized that Efp would also exhibit its action in endometrial cancer cells through modulating NF-κB signaling. To examine the effect of Efp on NF-κB-mediated transcription, we performed a luciferase assay in which Ishikawa and HEC-1A cells were transfected with a NF-κB-driven luciferase reporter plasmid together with the Efp expression plasmid or siEfps (Fig 7A–7D). In both of these cells, Efp overexpression increased NF-κB-mediated transcription, whereas treatment with siEfps decreased NF-κB-mediated transcription.

### Efp regulates NF-κB signaling in endometrial cancer cells

**Fig 8:**
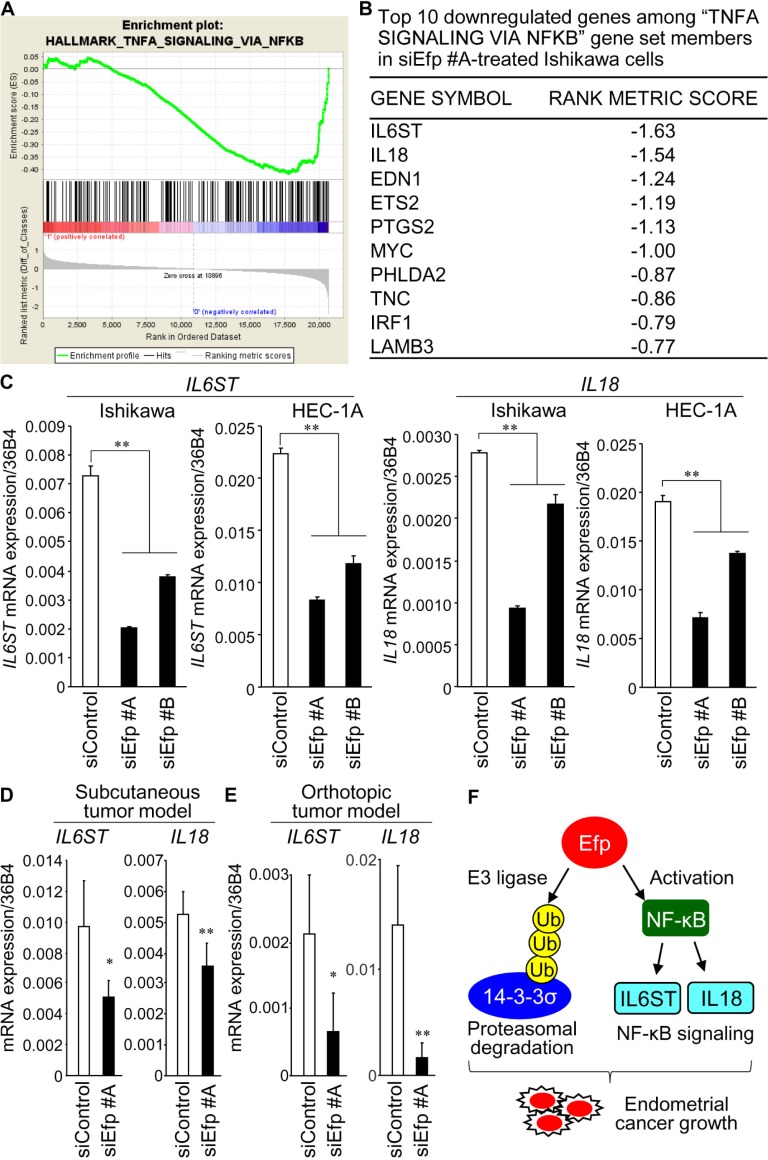
Gene set enrichment analysis revealed NF-κB signaling as a potential downstream regulator of Efp. (**A** and **B**) RNA from siEfp #A- or siControl-treated Ishikawa cells were subjected to microarray analysis and, subsequently, gene set enrichment analysis (GSEA) was performed using the GSEA software (https://software.broadinstitute.org/gsea/index.jsp). GSEA revealed that the gene set ‘TNFA SIGNALING VIA NFKB’ was enriched among the genes that had low expressions in siEfp #A-treated Ishikawa cells in the microarrays (A). Top 10 downregulated genes among “TNFA SIGNALING VIA NFKB” gene set members in siEfp #A-treated Ishikawa cells are shown (B). (**C**) Silencing of Efp decreases the expression of *IL6ST* and *IL18* genes. Ishikawa and HEC-1A cells were transfected with siEfp #A, #B, or siControl, and the expression levels of *IL6ST* and *IL18* mRNAs were quantified by qRT-PCR. Data are presented as means ± s.d. (*n* = 3). **, *P* < 0.01. (**D** and **E**) Decrease of *IL6ST* and *IL18* mRNA levels in siEfp #A-treated tumors *in vivo*. RNAs were isolated from the siEfp #A- or siControl-treated tumors generated in the subcutaneous inoculation experiment using Ishikawa cells shown in Fig 5(D) and orthotopic inoculation experiment using HEC-1A cells shown in Fig 6(E). The expression levels of *IL6ST* and *IL18* mRNAs were quantified by qRT-PCR. Data are presented as means ± s.d. (*n* = 3). *, *P* < 0.05; **, *P* < 0.01. (**F**) Model for the role of Efp in endometrial cancer. Efp could act as an E3 ubiquitin ligase to promote degradation of 14-3-3σ protein. Moreover, Efp is suggested to promote endometrial cancer by enhancing the NF-κB-mediated transcription and its donwnstream signals including IL6ST and IL18.

To explore the effects of siEfp on the gene expression profile of endometrial cancer cells, we performed microarray experiments to compare the gene expression in Ishikawa cells treated with siEfp #A or siControl. Subsequently, we performed GSEA to identify statistically significant gene sets showing differential expression between them. The siEfp #A-treated Ishikawa cells exhibited down- and up-regulated gene sets compared with siControl-treated cells (S1 Table). Notably, the downregulated gene sets contained NF-κB signaling (Fig 8A and 8B) and its associated pathway such as signal transducer and activator of transcription 3 (*STAT3*), *STAT5*, IFN-γ, and IFN-α. The siEfp #A and #B-mediated downregulation of interleukin 6 signal transducer (*IL6ST*) and interleukin 18 (*IL18*), both of which participate in NF-κB signaling, was validated by qRT-PCR analysis in Ishikawa and HEC-1A cells (Fig 8C). Moreover, expression of *IL6ST* and *IL18* was examined in the tumors generated in our subcutaneous and orthotopic mouse xenograft models shown in Figs 5 and 6, respectively. We found that mRNA levels of *IL6ST* and *IL18* were significantly decreased in the siEfp #A-treated tumors compared with the corresponding siControl-treated tumors both in the subcutaneous (Fig 8D) and orthotopic mouse models (Fig 8E).

## Discussion

In the present study, we showed that Efp knockdown inhibits the growth, cell cycle progression, and migration of endometrial cancer ER-positive Ishikawa and ER-negative HEC-1A cells. Using *in vivo* tumor model of athymic mice, the Efp-specific siRNA suppresses the proliferation of Ishikawa-derived subcutaneous tumors by direct injection and HEC-1A-derived orthotopic tumors by intravenous administration. Efp knockdown effectively represses Efp expression and increased the protein level of cell cycle checkpoint 14-3-3σ, which is a known substrate for Efp ubiquitin ligase. Moreover, Efp stimulates NF-κB-mediated transcription and modulates the expression of NF-κB-related genes in both Ishikawa and HEC-1 cells.

Functional analysis of Efp has been mainly performed in breast cancer and postulated its promotive role on tumor cell growth. Namely, Efp functions as an ubiquitin ligase that degrades 14-3-3σ protein via proteasome system [6]. A clinicopathological study showed that Efp immunoreactivity correlates with poor prognosis and is defined as an independent marker for patients with breast cancer [7]. Considering that Efp is a prototypic ER target gene, which possesses a functional estrogen-responsive element (ERE) in its 3’-untranslated region (UTR)[4], Efp expression may also depend on ER status in endometrial cancer. Although the majority of endometrial cancer is estrogen-related, only a few reports have been published in terms of the pathophysiological role of Efp in endometrial cancer. One report showed that estrogen upregulates Efp expression at both mRNA and protein levels in Ishikawa cells along with the increased cell growth [24]. The study also showed that Efp siRNA suppressed the growth of Ishikawa cells even in the presence of estrogen and decreased the estrogen-induced VEGF expression. Taken together, the present results together with previous studies support the notion that Efp functions a tumor-promoting factor for endometrial cancer cells. Another report showed that Efp mRNA expression is intense in G1 stage well-differentiated endometrial cancers, whereas it is repressed in G3 stage poorly-differentiated endometrial cancers [25].

In RL95-2 endometrial cancer cells, it has been shown that 14-3-3σ expression was increased when the cell growth was inhibited and arrested at G2/M phase of the cell cycle by 5-aza-2’-deoxycytidine (DAC) treatment [26]. Knockdown of 14-3-3σ stimulated endometrial cancer cell growth and impaired the effect of DAC on cell cycle arrest, whereas overexpression of 14-3-3σ inhibited cell growth and colony formation. Clinically, low immunoreactivity of 14-3-3σ was correlated with high risk of recurrence or death in endometrial cancer [27]. These findings suggest that Efp and 14-3-3σ could play a pivotal role in endometrial cancer.

Notably, our microarray experiment revealed that Efp silencing leads to downregulation of NF-κB pathway genes including *IL6ST* and *IL18*. IL6ST, also known as glycoprotein 130 (gp130), is a shared subunit of receptor complexes for cytokines including IL-6, leukemia inhibitory factor (LIF), and oncostatin M (OSM) [28]. NF-κB stimulates IL6ST-mediated signaling through the transcriptional regulation of target genes IL-6 and LIF [29]. IL6ST transduces signals concerning many biological processes including the immune response, inflammation, and embryonic development, whereas, in cancer, activation and dysregulation of IL6ST is considered to play a role in the progression of the disease [28]. Although IL6ST has been shown to activate several signaling pathways including STAT3, Janus tyrosine kinase (JAK), mitogen-activated protein kinase (MAPK), and phosphatidylinositol 3-kinase (PI3K), among them, STAT3 has been hypothesized to be a key downstream molecule in cancer [30]. Indeed, overexpression and constitutive activation of STAT3 have been found in multiple types of tumors, including endometrial cancer [31]. We speculate that Efp can modulate inflammatory signals that were closely related to the pathogenesis of tumors [32]. IL18 is a member of the IL-1 cytokine family and plays important roles in the immune system including the production of cytokines such as IFN-γ [33]. NF-κB participates in the IL18-induced IFN-γ expression [34]. In contrast, NF-κB also mediates IL18 expression in response to TNFα [35]. IL18 produced by immune cells has been shown to enhance immune responses. Namely, IL18 stimulated T helper 1 (Th1)-mediated immune responses through the induction of IFN-γ and promoted natural killer (NK) cell activation [36]. However, IL18 was also expressed in cancer cells and plays a distinct role in tumor pathophysiology. It was reported that higher plasma IL18 levels were associated with shorter recurrence-free survival (RFS) and overall survival (OS) in patients with triple-negative breast cancer [37], and IL18 overexpression in tumors was associated with shorter OS in patients with pancreatic cancer [38]. IL18 also enhanced the migratory ability of cancer cells including pancreatic, breast, and gastric cancers [39]. Moreover, tumor-derived IL18 has been demonstrated to increase the immunosuppressive NK cells in which programmed death 1 (PD-1) expression was induced [40]. Taken together, Efp is suggested to promote endometrial cancer by enhancing the signals of NF-κB and its related factors IL6ST and IL18 in addition to downregulation of 14-3-3σ protein, an Efp ubiquitin ligase-substrate (Fig 8F).

HEC-1A cells are often described as ERα-negative and exhibit the estrogen-independent cell growth [41]. HEC-1A cells also have characteristics of drug resistance against cisplatin and rapamycin [42, 43]. Thus, we propose that Efp could exert a tumor-stimulatory role in endocrine- and drug-resistant endometrial cancers as well as in estrogen-related cancers. Notably, our previous study showed that Efp expression is also regulated by other transcription factors besides ERα, as the 5’-flanking promoter region of Efp gene could be regulated by multiple elements such as an E-box near the transcription initiation site and its binding transcription factor, upstream stimulatory factor [44]. Indeed, we showed that siRNAs against Efp could significantly repress the growth of HEC-1A cells in this study, suggesting that the ERα-independent transcriptional regulation of Efp has a substantial impact on ERα-negative endometrial cancer. Vice versa, the ERα-independent Efp transcription may also play a critical role in ERα-positive endometrial cancer, as we showed that Efp siRNAs more substantially inhibit the growth of Ishikawa cells compared with ERα siRNAs even in the presence of estrogen.

Efp also have a critical function in the progression of other cancers. In ovarian cancer, Efp immunoreactivity was positively correlated with ERα/β immunoreactivities and was significantly higher in a subgroup with serous adenocarcinoma [45]. Overexpression of Efp was also shown in lung cancer specimens. In lung cancer cells, Efp is considered to stimulate the cell proliferation and migration by modulating p53 expression [46]. In gastric cancer, high levels of Efp mRNA expression were correlated with poor prognosis of patients [47]. This study showed that ectopic expression of Efp reversed the effects of TGF-β inhibitor on the migration and invasion of gastric cancer cells by increasing the expression levels of phosphorylated Smad2/4 and matrix metalloprotease-2 and -9, suggesting that Efp promotes cell migration and invasion by regulating the TGF-β pathway. Recently, Efp was reported as a novel regulator of p53 and Mdm2 [48]. Efp reduces p53 polyubiquitination by inhibiting the association of p300 with Mdm2 that is required for p53 polyubiquitination. Although Efp increases p53 abundance, p53 activity is assumed to be decreased by Efp since Efp may repress the acetylation of p53 and p53-dependent cell death. In addition, we previously demonstrated that Efp promotes cell proliferation and survival in prostate cancer by promoting GTPase-activating protein-binding protein 2 (G3BP2)-mediated nuclear export of p53 protein [49]. These observations imply that Efp would be a promising candidate for a molecular target for the treatment of cancers. Overall, Efp would exert a tumor-promoting role in endometrial cancer by association with several signaling pathways including NF-κB.

## Supporting information

S1 Table Down- and up-regulated molecular signatures in siEfp #A-treated Ishikawa cells. (DOCX)

S1 Fig Efp silencing decreases growth of Ishikawa cells regardless of estrogen treatment. (A) Knockdown efficiency of ERα siRNAs (siERαs) in Ishikawa cells. Cells were transfected with siERα #A, #B, or negative control siControl for 48 h. ERα mRNA levels were determined by qRT-PCR and normalized to *36B4* levels. (B) Growth inhibition of Ishikawa cells transfected with siRNAs targeting Efp and ERα. Ishikawa cells were transfected with the combinations of siRNAs: siEfp #A, siEfp #B, siERα #A, siERα #B, or siControl, as indicated, and cultured in the presence or absence of 10 nM 17-β estradiol (E_2_). The WST-8 cell proliferation assay was performed at day 5 after the transfection. The absorbance of the plates was read on a microplate reader at 450 nm. Data are presented as means ± s.d. (*n* = 3). **, *P* < 0.01. ns, not significant.
(EPS)
