# Supplementary material for: Efp promotes in vitro and in vivo growth of endometrial cancer cells along with the activation of nuclear factor-κB signaling
Source: PLoS One. 2018 Dec 26;13(12):e0208351. doi: 10.1371/journal.pone.0208351 (PMC6306158; doi:10.1371/journal.pone.0208351)
Supplement: S1 Table — (DOCX) [file pone.0208351.s001.docx]

S1 Table. Down- and up-regulated molecular signatures in siEfp #A-treated Ishikawa cells.

| Molecular Signatures | | NES^a)^ | NOM *p*-val^b)^ |
| --- | --- | --- | --- |
| Downregulated | |  |  |
|  | HALLMARK_MYC_TARGETS_V2 | -2.11 | < 0.01 |
|  | HALLMARK_EPITHELIAL_MESENCHYMAL_TRANSITION | -2.05 | < 0.01 |
|  | HALLMARK_IL6_JAK_STAT3_SIGNALING | -1.91 | < 0.01 |
|  | HALLMARK_UV_RESPONSE_DN | -1.87 | < 0.01 |
|  | HALLMARK_ESTROGEN_RESPONSE_EARLY | -1.84 | < 0.01 |
|  | HALLMARK_COAGULATION | -1.82 | < 0.01 |
|  | HALLMARK_TNFA_SIGNALING_VIA_NFKB | -1.77 | < 0.01 |
|  | HALLMARK_ANDROGEN_RESPONSE | -1.71 | < 0.01 |
|  | HALLMARK_INTERFERON_GAMMA_RESPONSE | -1.68 | < 0.01 |
|  | HALLMARK_IL2_STAT5_SIGNALING | -1.62 | < 0.01 |
|  | HALLMARK_APICAL_JUNCTION | -1.55 | < 0.01 |
|  | HALLMARK_APOPTOSIS | -1.55 | < 0.01 |
|  | HALLMARK_HYPOXIA | -1.50 | < 0.01 |
|  | HALLMARK_INTERFERON_ALPHA_RESPONSE | -1.48 | 0.019 |
|  | HALLMARK_HEDGEHOG_SIGNALING | -1.45 | 0.044 |
|  | HALLMARK_TGF_BETA_SIGNALING | -1.41 | 0.047 |
| Upregulated | |  |  |
|  | HALLMARK_REACTIVE_OXIGEN_SPECIES_PATHWAY | 1.40 | 0.049 |
|  | HALLMARK_OXIDATIVE_PHOSPHORYLATION | 1.27 | 0.023 |

a) Normalized enrichment score

b) Nominal *p*-value
